# Dietary whey protein lessens several risk factors for metabolic diseases: a review

**DOI:** 10.1186/1476-511X-11-67

**Published:** 2012-07-10

**Authors:** Gabriela TD Sousa, Fábio S Lira, José C Rosa, Erick P de Oliveira, Lila M Oyama, Ronaldo V Santos, Gustavo D Pimentel

**Affiliations:** 1 Departamento de Fisiologia e Biofísica, Instituto de Ciências Biomédicas, Universidade de São Paulo (USP), São Paulo/SP, Brazil; 2 Laboratório de Bioquímica e Fisiologia do Exercício, Universidade do Extremo Sul Catarinense, Criciúma/SC, Brazil; 3 Departamento de Patologia, Universidade Estadual Paulista (UNESP), Botucatu/SP, Brazil; 4 Departamento de Fisiologia, Universidade Federal de São Paulo (UNIFESP), São Paulo/SP, Brazil; 5 Departamento de Psicobiologia, Universidade Federal de São Paulo (UNIFESP), São Paulo/SP, Brazil; 6 Departamento de Clínica Médica, Universidade Estadual de Campinas (UNICAMP), Campinas/SP, MA:, 13083-970, Brazil

**Keywords:** Whey protein, Obesity, Type 2 diabetes mellitus, Appetite, Inflammation, Hypertension.

## Abstract

Obesity and type 2 diabetes mellitus (DM) have grown in prevalence around the world, and recently, related diseases have been considered epidemic. Given the high cost of treatment of obesity/DM-associated diseases, strategies such as dietary manipulation have been widely studied; among them, the whey protein diet has reached popularity because it has been suggested as a strategy for the prevention and treatment of obesity and DM in both humans and animals. Among its main actions, the following activities stand out: reduction of serum glucose in healthy individuals, impaired glucose tolerance in DM and obese patients; reduction in body weight; maintenance of muscle mass; increases in the release of anorectic hormones such as cholecystokinin, leptin, and glucagon like-peptide 1 (GLP-1); and a decrease in the orexigenic hormone ghrelin. Furthermore, studies have shown that whey protein can also lead to reductions in blood pressure, inflammation, and oxidative stress.

## Introduction

Excess bodyweight in humans has been increasing worldwide. It is considered an epidemic by the World Health Organization (WHO) [1,2]. Recently, it was estimated that more than 300 million people worldwide are obese and more than 1 billion are overweight [3].

Similar to obesity, type 2 diabetes mellitus (DM) has been regarded as a major global epidemic of the 21^st^ century [4]. In addition, obese individuals twice as likely to develop metabolic syndrome (MS) comorbidities [5].

Obesity and DM are MS-associated diseases. Although the pathogenesis of MS and each of its components is complex and poorly understood, abdominal obesity and insulin resistance are recognized as risk factors for MS. Furthermore, patients with MS have a five-fold higher risk of developing DM [6].

Patients with DM have an elevated response to postprandial triacylglycerols compared to non-diabetic subjects. Additionally, postprandial triacylglycerols are also known to be strongly associated with cardiovascular diseases [7].

Reductions in body weight can reduce obesity-related problems [8-10]. Accordingly, dietary manipulations may promote increased satiety, to stimulate the anorexigenic hormones and consequent to reduce food intake and body weight [11]. Therefore, is extremely important to discover strategies that maximize the effect of weight loss and inhibit weight regain after short- and long-term of nutritional counseling [2,12,13]. Likewise, diet supplementation with milk serum protein has been suggested as an adjunct strategy in the prevention and treatment of obesity and MS-related diseases in humans [14,15] and animals [16,17]. In addition, dietary milk serum proteins, e.g., whey protein, have high nutritional value because it contains all essential amino acids in higher concentrations than vegetable protein sources [18,19]. Based on that, this review aims to discuss the main effects of whey protein in the treatment or prevention of obesity, DM, hypertension, oxidative stress and MS-linked metabolic complications.

## Methodology

For the preparation of this review, we performed bibliographic searches in databases of the CAPES Periodic Portal, Scielo, and Medline/Pubmed, covering articles published between 2003 and 2012. A search of articles was made using the key words “whey protein,” “milk serum protein,” “type 2 diabetes mellitus,” “obesity,” “insulin resistance,” “inflammation,” “hypertension,” “metabolic syndrome,” and “protein hydrolysates.”

### Main nutritional and functional components of whey protein

Milk serum proteins are defined as substances that remain soluble in milk serum [20]. These proteins are naturally formed during the production of cheese [19,21] and account for 20% of the all protein in milk [21-23], such as β-lactoglobulins, α-lactalbumin, immunoglobulins, lactoferrin, lactoperoxidase, glycomacropeptide, bovine serum albumin [18,20,22,24], and other proteins [22].

In addition, whey protein has high concentrations of branched chain amino acids (BCAAs), such as leucine, isoleucine, and valine, which are also related to important factors for muscle growth, build, and repair [14,25].

Milk serum proteins do not coagulate in acidic conditions; they resist the action of quimosine from the stomach, quickly reach the jejunum [19], are rapidly digested, and raise plasma amino acid concentrations of [21,26]. Therefore, milk serum proteins perform several functions, such as mineral absorption, improvement of protein synthesis, sensitivity to hormones, and decreased blood glucose and lipid levels [7,15,16,21,23,27-29]. In summary, the main nutritional and functional components of whey protein are presented in Table 1.

**Table 1:** Main components and actions of whey protein

**Components**	**Actions**
β-lactoglobulin (45–57%)	Has content higher of branched chain amino acids (~25.1%). Capture hydrophobic molecules, participating in the reduction of intestinal absorption of lipids.
α-lactalbumin (15–25%)	Has content higher of tryptophan (6%) of all dietary proteins. It is rich in lysine, leucine, threonine, and cysteine. It has the ability to bind to minerals such as Ca and Zn, positively affecting their absorption.
Immunoglobulin (10–15%)	Four classes of immunoglobulins are present in serum: IgG, IgA, IgM, and IgE. It functions as an antioxidant protection and increases immunity.
Lactoferrin (~1%)	Inhibits the production of pro-inflammatory cytokines and protects against the development of hepatitis.
Lactoperoxidase (<1%)	Important antimicrobial properties
Glicomacropeptide (10–15%)	It is formed from the digestion of κ-casein during coagulation of cheese. It is high in essential amino acids that favor the absorption of minerals.
Bovine serum albumin	Good profile amino acid and function of binding to lipids.

Muro Urista et al. [30], Graf et al. [31], Gilbert et al. [32], Dougkas et al. [33], Madureira et al. [34].

### Improvement risk factors for metabolic diseases

#### Obesity

Several types of diets are being studied to find a model that has the quality and quantity of nutrients to promote weight loss, reduction of body fat, maintenance of muscle mass and satiety control [10,13,14,29,35-37].

Frestedt et al. [10] demonstrated that the supplementation with a mixture of whey protein isolate with other peptides (2 servings/day and each serving with 10 g protein) for 54 healthy subjects for 12 weeks, in addition to 47 control subjects who consumed glucose (10 g/serving) led to weight loss in both groups, but the group supplemented with whey protein had higher weight loss. They also had greater reductions of body fat (6.1%) and a higher maintenance of lean mass than the control group who had consumed just glucose.

Pilvi et al. [13] studied mice that had diet-induced obesity and were subsequently fed a low-calorie diet with different types of proteins (whey protein isolate, α-lactalbumin, β-lactoglobulin, and lactoferrin) for 50 days, and after this period, they returned to high fat diet. The mice that consumed α-lactalbumin (45.9 g) had a significant loss of fat mass during the caloric restriction period and a reduction in visceral fat during the weight recovery period when compared to other groups. But this is not a conclusive explanation about the mechanisms that lead to such results.

Another component of milk, calcium, has been extensively studied with a possible anti-obesity role. But Pilvi et al. [29] observed a significant decrease in weight gain and body fat and a higher fat excretion in mice fed for 21 weeks with high-fat diet (60% of total calories from fat), 18% protein (whey protein isolate), and 1.8% calcium carbonate (CaCO_3_) in comparison with a similar diet that had casein as the protein source. The authors suggest that a possible explanation for this result is the high levels of leucine present in the whey protein, e.g., the leucine may modulate insulin signaling by phosphatidylinositol 3 kinase (PI3K) directing the energy to muscle synthesis and not for storage in adipose tissue. Subsequently, Pilvi et al. [38] showed that mice that consumed a high-fat diet (60% of total calories from fat) with 18% protein (whey protein isolate) and 1.8% calcium carbonate for 12 weeks had not only an inhibition in the accumulation of fat mass but also an increase in gene expression in the visceral adipose tissue of leptin and β3-adrenergic receptor when compared to another group of mice that received a diet with similar fat and protein contents, that instead used casein instead of whey and only 0.4% calcium carbonate. Thus, the authors suggest that the whey protein isolate may reduce obesity via improvement of leptin sensibility.

Baer et al. [35] showed that the type of protein can influence the weight loss response. Supplementation with 56 g of whey protein (consumed twice daily) for 23 weeks diminished body weight and fat mass when compared with the group that consumed just carbohydrates. Moreover, waist circumference and fasting ghrelin levels were lower in the whey protein group when compared to the group that ingested soy protein. These results demonstrated that through yet-unknown mechanisms, different sources of dietary protein may differentially facilitate weight loss and affect body composition.

In addition to the type dietary of protein, the proportion of nutrients also influences energy consumption and body composition [37]. Pichon et al. [37] observed a reduction in body weight and adipose tissue in rats after 25 days of consuming a high-protein diet (55% of total energy intake) compared to rats that consumed a normal protein diet (14% of energy). In addition, the results were more pronounced with the high protein diet containing whey protein isolate enriched with β-lactoglobulin compared with other protein sources, such as whole milk and whey protein concentrate. The authors suggested that these results were obtained because the proteins have a greater power of satiety when compared to the other nutrients. Moreover, β-lactoglobulin may capture hydrophobic molecules, decreasing the absorption of fat by intestinal cells. Furthermore, a high protein diet induces to increases in thermogenesis [20].

Furthermore, the reduction of body weight and body fat [9] and the decrease of serum triacylglycerols levels in obese individuals are extremely important, since these individuals are at increased risk for cardiovascular diseases [23,26]. Recently, Mortensen et al. [7] showed a decrease in triacylglycerol levels when whey protein was supplemented in DM patients. In addition, Pal et al. [23] confirmed these results in both overweight and obese post-menopausal women. This reduction was found after consumption of 45 g of whey protein isolate together with a high-fat meal when compared to consumption of the equivalent amount of glucose or casein. Pal et al. [39] also showed a decrease in triacylglycerol concentration in both overweight and obese individuals after 12 weeks of supplementation with 54 g of whey protein compared to control group (without supplementation) and with consumption of the same amount of glucose. As a reduction of ~20% in serum triacylglycerols has been shown to reduce the progression of coronary diseases, these results are very important to decreasing obesity.

Kasim-Karakas et al. [40] studied the influence of the ingestion of whey protein in overweight and obese women with polycystic ovary syndrome (PCOS). It has been known that a reduction in body weight improves the symptoms of PCOS. Accordingly, the intake of 75 g of whey protein isolate compared to 75 g of glucose reduced the ghrelin levels for 5 hours after consumption. Therefore, these results also suggest that the whey protein can prolong satiety.

Recently, Pal and Ellis [41] showed in overweight and obese individuals that supplementation of whey protein (54 g) for 12 weeks did not significantly reduce the body weight, BMI, waist circumference, and total body fat, but decreases the triacylglycerol and insulin levels after treatment.

Collectively, these studies that relationship whey protein and obesity showed improvement in insulin sensitivity and lipid profile with possible increase of energy expenditure.

#### Type 2 diabetes mellitus

Although insulin is a hormone anorectic [42-45] and suppresses ghrelin [40], it is also an anabolic hormone and therefore is related to increases in muscle protein synthesis [42]. Furthermore, hyperinsulinemia inhibits hormone sensitive lipase (HSL), suppressing the release of fatty acids from adipose tissue [7] and stimulates the lipoprotein lipase (LPL) and fatty acid synthesis, contributing to obesity [40]. However, the increase in insulin levels after consumption of whey protein reported in several studies is not able to promote the increase of fat mass, perhaps due to the high leucine contents present in whey protein and also because its consumption over the long-term (>12 weeks) improves insulin sensitivity [41].

Recent studies have shown the important role of whey protein supplementation in glycemia control, possibly through the stimulation of incretin hormones, which increase fasting and postprandial insulin release and improve insulin sensitivity [41,46-48].

Pichon et al. [37] showed that a high-protein diet raises insulin concentrations compared to a normoproteic diet. However, in human and rat studies in which the protein is combined with carbohydrate, an increase in the insulin response has been observed [14-16]. In addition, whey protein stimulates insulin secretion, and when compared to casein, milk serum proteins have increased postprandial insulinotropic effects that are probably mediated by the rapid serum absorption of BCAAs, the improvement in glucose homeostasis in DM, and the possible delay or withdraw of the medicine [46] .

Recently, Gunnarsson et al. [16] showed the effects of acute administration of whey protein plus glucose by nasogastric tube (enteral diet) in mice when compared to the administration of only glucose. In this study, the authors found an increase in insulin levels three times greater and an insulin tolerance four times greater with consumption of whey protein.

Petersen et al. [15] observed in healthy subjects a significant reduction in postprandial glucose (37.5%) when consumed in a single dose containing 50 g of carbohydrate plus milk serum proteins (20 g in total). This decrease was dose-dependent; thus, the higher the protein intake, the greater its effect on blood glucose. The same effect was observed by Frid et al. [46] in DM subjects that consumed milk serum protein. In this study, the authors found a significant increase in insulin and glucose-dependent insulinotropic peptide (GIP).

Recently, Mortensen et al. [7] confirmed the hypoglycemic effect of whey protein in individuals with DM within eight hours of consuming a meal test that contained 45 g of whey protein compared to three other meals containing different protein sources, casein, gluten, and codfish. Moreover, within six hours of a meal test a reduction in triacylglycerol concentrations was observed with the supplementation of whey compared to three other meals that contained a different type of protein. Interestingly, all protein meals were associated with a high-fat diet (100 g of butter).

Lan-Pidhainy and Wolever [28] also observed a significant hypoglycemic effect in individuals with insulin resistance after the consumption of a drink containing 30 g of whey protein plus 50 g of glucose compared to groups of individuals who consumed only 50 g glucose or another group who consumed 50 g of glucose plus 30 g of canola oil.

Studies performed in rats [16,17] showed that supplementation with whey protein possibly suppressed serum glucose level by the inhibition of the enzyme dipeptidyl peptidase-IV (DPP-IV), whose function is to disable the incretin hormones, such as GLP-1 (glucagon-like peptide 1) and GIP, which are both related to glycemic control. Gunnarsson et al. [16] suggest, in mice, that the digestion of whey protein leads to the formation of di- and tri-peptides that are a substrate for DPP-IV. Nevertheless, Frid et al. [46] and Mortensen et al. [7] found no changes in blood GLP-1 levels, only decreased glucose levels after consumption of different amounts, 36.4 g and 45 g of whey protein in DM individuals. These findings may possibly occur because, in diabetic subjects, the secretion postprandial GLP-1 is decreased [15,49] and the enzyme activity DDP-IV is increased [50].

In healthy individuals, an increased GLP-1 levels due to consumption of whey protein is more palatable [51]. Recently, Veldhorst et al. [51] observed an increase in blood insulin (91%) and GLP-1 (164%) levels after consumption of a diet containing 25% of calories from protein (whey protein), 55% from carbohydrates, and 20% from lipids compared to similar quantities of casein. In addition, non-significant reduction of blood glucose with the consumption of whey compared to casein was observed.

In summary, protein is important in fetal growth and development of the pancreas. Furthermore, adaptations to nutritional stress may permanently alter the physiology and metabolism of several organs, leading to long-term diseases such as cardiovascular diseases, DM, and MS [24]. Likewise, Barnett et al. [24] observed reduction of 55–65% in insulin secretion in adult life in the offspring of mother rats who consumed low amounts of protein (whey protein) during pregnancy. This reduction is related to the early development of DM in adult humans.

Therefore, whey protein may be utilized by reduce insulin resistance due the increase in secretion of GLP-1 and to reduce serum glucose and insulin levels.

#### Hypertension

Hypertension is commonly found in patients with DM and may affect approximately 60% of Brazilian individuals [52].

Recently, it was discovered that diet is a major determinant of blood pressure. Likewise, certain foods have a direct role in the reduction of blood pressure or additional reductions in cardiovascular mortality [41,53]. Some amino acids of the whey protein, e.g., α-lactalbumin and β-lactoglobulin, are precursors of peptide inhibitors of angiotensin-converting enzyme (ACE) [20]. ACE is a key enzyme in the regulation of blood pressure [41].

Pal and Ellis [41] showed the hypotensive effect that occurs after intake of whey protein (54 g protein) and casein (27 g protein) in either obese or overweight normotensive individuals. However, the components of proteins that could possibly lead to the improvement of blood pressure were not analyzed in this study. Likewise, Lee et al. [54] found no decrease in blood pressure in individuals with mild hypertension who consumed a drink containing skim milk with milk serum proteins (125 mL) for 12 weeks. The low level of peptides (2.6 g per 100 g of protein drink) administered together with protein may have been responsible for the absence of an effect on blood pressure.

In summary, whey protein is associated with reduction blood pressure by inhibition of ACE enzyme and possibly via lower body weight gain in individuals that habitually consumed the aminoacids from whey protein or BCAA than those subjects that consumption others aminoacids, for e.g. non essential. Therefore, whey protein can in the future be considered extremely important for the control of hypertension.

### Possible mechanisms involved in reducing risk factors for metabolic diseases

#### Reduction of food intake

Satiety is an important factor in the regulation of food intake and also in the control of obesity [11,45]. Dietary protein and specific amino acids are involved in the control of gastric and intestinal motility and in pancreatic secretion, and are more potent in inducing satiety than carbohydrates or fats [55].

Gut peptides that regulate the digestive process and neuronal signaling in the central nervous system (CNS) regulate hunger and satiety [42]. Table 2 lists several peripheral hormones and their roles in the regulation of food intake [57].

**Table 2:** Characteristics and functions of hormones related to appetite regulation

**Hormones**	**Production and effects**
CCK	Produced: duodenum
	Effect: reduces appetite
GLP-1	Produced: mainly in distal intestine (L cells)
	Effect: reduces appetite
Ghrelin	Produced: stomach
	Effect: stimulates appetite
GIP	Produced: K cells of the duodenum
	Effect: reduces appetite and potentiate insulin release
Leptin	Produced: mainly in adipose tissue
	Effect: suppress appetite
Uroguanylin [56]	Produced: intestinal epithelial cells
	Effect: reduces appetite

CCK: cholecystokinin, GLP-1: glucagon-like peptide 1, GIP: glucose-dependent insulinotropic peptide. Adapted: Pimentel & Zemdegs, 2010 [57] and Pimentel et al. [58].

Milk serum proteins are more potent stimulants of cholecystokinin (CCK) and GLP-1 than casein [26,41,51]. Among the peptides involved with whey protein, glycomacropeptide is an effective secretagogue of CCK [36]. CCK is a hormone secreted by I cells of the small intestine that has as one of its functions to modulate satiety [59]. However, Burton-Freeman [36] did not observe the effect of dietary whey protein on the increase of postprandial CCK levels in healthy subjects. Therefore, we may to infer that probably a dose of whey was insufficient to stimulate the CCK.

Pal and Ellis [41] observed a significant decrease in glucose, appetite, and food intake, and an increase in serum insulin levels after the consumption of a drink containing 50 g of whey protein when compared to the consumption of a similar amount of protein tuna, turkey, or egg albumin. This study suggests a potential application of these foods in appetite control in both overweight and obese individuals.

Furthermore, other amino acids not described in this review are also associated with satiety, such as tryptophan, which is a precursor of serotonin and an important modulator of appetite [51].

Although the gut hormones are known by increase of anorexigenic hormones, also was observed that the leucine when injected directly in the central nervous system reduces the food intake and body weight [60]. Furthermore, Ropelle et al. [61] found that both leucine intracerebroventricular injection or high-protein diet decrease AMPK and increase mTOR phosphorylation in the CNS inhibiting the neuropeptide Y and stimulating the pro-opiomelanocortin expression, leading to reduction of food intake.

In summary, these findings suggesting that the aminoacids from whey protein may reduce the food intake via increase of gut hormones (CCK and GLP-1), and reduction of neuropeptide orexigenic (NPY) and increase of neuropeptide anorexigenic (POMC) in the hypothalamus.

#### Anti-inflammatory actions

Adipose tissue is an endocrine organ that releases hormones, cytokines, and others substances that tend to impair insulin sensitivity [55]. Obese individuals have increased secretion of adipocytokines by adipose tissue and macrophages [20].

Recently, Pal & Ellis [41] observed in overweight and obese subjects that the supplementation of whey protein (54 g) for 12 weeks did not change the pro-inflammatory markers (IL-6, C-reactive protein-CRP, and TNF-α). However, in D-galactosamine-induced hepatitis and liver fibrosis in rats, the consumption of whey protein strongly reduced the plasma levels of pro-inflammatory cytokines (IL-1 beta: 59% and IL-6: 29%) compared to the consumption of the same amount of casein [62].

Collectively, reduction of pro-inflammatory cytokines may be associated with reduction of body weight gain after consumption of whey protein and it aminoacids.

#### Anti-oxidative stress actions

Oxidative stress has been associated with MS, which is a disease recognized by inflammatory effects that are linked with the activation of reactive oxygen species (ROS) [63-65]. Nowadays, indicators that are more typically used in the evaluation of ROS are the endogenous antioxidant enzymes such as glutathione peroxidase, catalase, and superoxide dismutase, and other components such as malondialdehyde (MDA) and thiobarbituric acid reactive substances (TBARS) [63-66]. Recently, the administration of 100 mg/kg of body weight of whey protein in streptozotocin-induced diabetic rats was found to decrease several oxidative stress indicators, such as MDA, nitric oxide, and ROS concentrations; as well as reduction of pro-inflammatory cytokines (IL-1β, TNF-α, IL-6 and IL-4) and increase glutathione levels [67]. Another study observed that rats fed high-carbohydrate, fat-free diets to induce fatty livers (nonalcoholic fatty liver model) plus orally administered whey protein (0.15 g/d/rat) for 28 days reduced MDA and increased glutathione levels [68].

Haraguchi et al. [19] found a protective effect against oxidative stress, mainly in the liver, and a beneficial effect on renal function in rats supplemented with whey protein plus a hypercholesterolemic diet, but they did not observe a reduction in serum cholesterol levels.

In human studies, beneficial effects in the reduction of oxidative stress after treatment with whey protein [69-71]. Likewise, supplementation with 20 g/d of whey protein isolate for 12 weeks in subjects with nonalcoholic steatohepatitis was found to increase the glutathione and total antioxidant capacities [70]. In healthy individuals, 45 g/day of whey protein supplementation in bar format for 14 days also increased lymphocyte glutathione levels [69]. De Aguilar-Nascimento et al. [71] studied patients with ischemic stroke that were fed via a nasogastric tube a diet with 35 kcal/kg/d and 1.2 g of protein/kg/d containing whey protein and an observed reduction in IL-6 and an increase in glutathione levels after five days of supplementation.

Furthermore, several studies also shown in different models of oxidative stress that only whey protein or diets that contain this protein improve antioxidant function and decrease oxidative stress [72-75].

Collectively, these findings suggest that whey protein may act as a nutritional component to increase endogenous antioxidant enzymes (glutathione peroxidase, catalase, and superoxide dismutase) and to reduce oxidative stress markers (MDA, TBARS) jointly with low expression of pro-inflammatory cytokines (IL-1β, IL-6 and TNF-α) in obese, diabetic or stroke patients.

### Commercialization and safety doses of whey protein

Whey protein can be found in drinks, powder, protein bars, and milk. The main natural source of whey protein is bovine milk that has approximately 3.5% protein, of which 80% is casein and the remaining 20% is whey protein [22,76]. Whey protein concentrate also may include 29–89% milk serum protein, and isolates should contain more than 90% whey protein [22]. In addition, whey protein can also be found in the form of hydrolysates. This form of whey protein hydrolysates aims to optimize the physical, chemical, and nutritional properties, improving the absorption of proteins [25].

According to the studies presented in this review, the amount of whey protein administered to adult humans is between 5.0–54.0 g at durations of approximately 12 weeks. Furthermore, no serious adverse effects were observed with whey protein supplementation. However, this supplementation must make part of a habitual diet.

### Future perspectives

**Figure 1:**
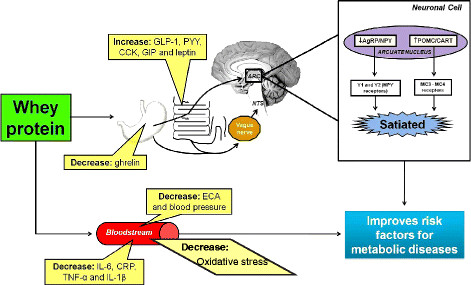
Main mechanisms of action of whey protein in protection of risk factors for metabolic diseases, such as obesity, type 2 diabetes mellitus, hypertension, oxidative stress and metabolic syndrome.

This review shows that whey protein may improve several risk factors for DM, obesity, hypertension, oxidative stress and MS (Figure 1). In addition, new studies suggest a relationship between consumption of whey protein source foods and oxidative stress, hepatoprotective effects, and increased resting energy expenditure.

Kume et al. [62] demonstrated a hepatoprotective effect of consumption of whey protein D-galactosamine-induced hepatitis and liver fibrosis in rats. There was a significant decrease in the activity of hepatic enzymes (AST: 92.5%, ALT: 98%, LDH: 65%, hyaluronic acid: 60%) after consumption of whey protein compared to casein.

In trained subjects, Hackney et al. [27] observed a significant increase (5%) after 24 hours of resting energy expenditure with the consumption of 18 g of whey protein before of a single session of resistance training (70–75% of one repetition maximum) when compared to an intake of 19 g of carbohydrates. The authors speculate that this increase occurred by the increased availability of amino acids to skeletal muscle after whey protein intake. In addition, there was a decrease in respiratory coefficients in both the consumption of carbohydrates (5%) and whey (6%); this reduction indicates the increased oxidation of fat. Furthermore, more studies are needed to determine whether supplementation of whey protein plus a balanced diet and resistance training can increase the long-term increase in resting energy expenditure and improve body composition.

## Conclusions

In summary, whey protein has an attractive effect on glucose metabolism control in healthy, overweight, obese, and insulin-resistant subjects. Moreover, whey protein assures a higher satiety; this effect is involved with the modulation of several gut hormones related to the reduction of food intake, with increased release of anorectic hormones, such as cholecystokinin, leptin, and GLP-1 and decreased release of the orexigenic hormone, ghrelin; and reduction of neuropeptide Y and increase of pro-opiomelanocortin in CNS. In addition, the reductions of expression of both inflammatory and oxidative stress markers, as well as the reduction in blood pressure, are also the main beneficial of risk factors for metabolic diseases.

## Competing interests

The authors declare that they have no competing interests.

## Authors’ contributions

GDP was the responsible by design of whole manuscript, GTDS and GDP wrote the paper, FSL, JCR, EPO, LMO, and RVS participated of choose and discussion of papers included . All authors read and approved the final manuscript.
